# Dietary in Disease

**Published:** 1864-10

**Authors:** Edward Smith

**Affiliations:** Assistant-Physician to the Hostal for Consumption and Diseases of the Chest, Brompton


### DIETARY IN DISEASE.

By EDWARD SMITH, M.D., F.R.S., F.R.C.P., Assistant-Physician to the Hostal for Consumption and Diseases of the Chest, Brompton.

I purpose, in a short series of papers, to state, in the most concise manner, the views which I entertain, and the grounds of them, in reference to the most suitable dietary in certain conditions of disease; and, in order to avoid repetition and misconception, I shall premise two general observations—viz.: that as in cases of disease there are degrees in severity and progress as well as certain exceptional conditions, I purpose to refer to those which present the usual symptoms, and have the fully developed effects of the disease, but are nevertheless in a remediable stage; and also that I do not intend to consider the pathology of disease further than may be necessary to establish bases for treatment. It will also be impossible for me to discuss at length the arguments by which any particular statement may be supported, but must be content to refer to the works of authors of the best repute, and to my own papers in the "Philosophical Transactions," or the separate works on the "Cyclical Changes" and on "Consumption in its early and remediable Stages."

### I. Diabetes.

*Wasting is the central condition of diabetes, around which all the other symptoms which mark the progress of the disease group themselves.*

This is not due to defect of ingesta, for, contrary to other states of wasting, the appetite for fluid and solid food is increased; the egesta are also increased, both absolutely and relatively to the ingesta.

The wasting of the body must be almost exclusively of the soft tissues, and hence must be of fluid (seeing that nearly 80 per cent. of the weight of the body is water) and fat and other products of tissue-waste. In this disease the wasting is—

1st. Of fluid, and that only through the kidneys; for the skin, lungs, and bowel emit rather less than in health.

2d. Of sugar, either eaten as sugar ready formed, or produced in the alimentary canal from starch which had not been converted into fat and deposited in the tissues, or into carbonic acid and emitted by the lungs; and in the absence of starch, from the animal tissues.

3d. Of urea, the chlorides and ather salts; for whilst the quantity in each ounce of urine is lessoned, the total amount emitted daily in the stage of disease preceding extreme emaciation is increased.* When much nitrogenous food is given, urea is further augmented in quantity, and may then be more the product of food than of tissue.

4th. Of fat, which is used by the function of respiration.

Let us consider each of these as indications of treatment.

1. *The fluid.*—Without going beyond our depth, we account for the dryness of the skin and fæces by the excessive elimina-

* In a case which ended fatally the quantity of urea emitted daily in January, was found by me to be as follows: 665, 624, 561, 700, 650, 650, 816, 792, 825, 729, 552, 540, 540, 610, and 533 grains.

tion of water by the kidneys, since the same correlation is observed daily in both health and disease. The thirst is consequent upon the excessive excretion of water, as it is under the influence of excretion or heat in health. Hence to diminish the latter and increase the former we must lesson the excessive excretion of water by the kidneys. The excretion is increased by the ingestion of fluids, whether separated or combined with solids; and so long as the latter is uncontrolled it will support or increase the former. In scarcely any case of diabetes is the quantity of ingested fluid recorded; in exceedingly few has it been limited, and where limited the restriction has been extreme, and the quantity allowed has been below that needful in health—as, for example, a pint and a half of fluid. Yet nothing has been more clearly proved than that the emission of fluid from the body is increased or diminished by the imbibition of it, and that a certain supply is necessary for the due peformance of vital actions. Hence the quantity must be limited; but not in a sudden manner, since the circulation has in a degree become accustomed to this large ingestion and emission of fluid, and to suddenly diminish the volume of the blood might arrest all vital actions; but by degrees and steadily, until it shall scarcely exceed, but be not less than, that in health—as, for example, in the fluids 3¾ lbs. (3 pints), and in the solids 1½ lbs.

*What relation has the sugar to the water?* Excessive elimination of water alone is more easily controlled than when sugar is largely present with it. Hence are both due to some anterior and common cause? or is the sugar a diuretic agent? In my experiments upon myself, in health, I found sugar to promote diuresis when taken with water only on an empty stomach in the morning; and although this was not always the case, the effect was so frequent that in the treatment of diabetes the mutual relation of the water to the sugar should be so regarded, as that water is necessary to the emission of sugar, and sugar is promotive, within limits, of the emission of water. Hence another reason for limiting the ingestion of water, and another explanation of the partial inefficacy of the plan; for if sugar be a diuretic, it will abstract fluid from the tissues if there be none to spare in the blood, and thus increase waste.

*What relation have the urea and salts to the water?* There is no evidence to show that urea and the chlorides are diuretic, yet they require water for their elimination; and *cœteris paribus*, as the water is increased daily so also (but not necessarily in proportion) will be the excretion of the urea and salts. Hence another reason for controlling the ingestion of water.

2. *The sugar.*—The daily ingestion of separated and combined sugar in health is from two to four ounces; but sugar is produced in the primary transformations of starch within the body by the action of the saliva and the pancreatic juice, and also (in however small or large a proportion) from the animal tissues. Both classes of foods agree in furnishing carbon, hydrogen, and oxygen, as carbonic acid and water, and perhaps free hydrogen, in their transformations, and differ chiefly in that flesh contains nitrogen and a larger proportionate quantity of fluid, and is eaten in much less amount than starchy foods. Hence, if for no other reasons, starchy foods will be the chief sources of sugar; but when flesh and other animal foods only are eaten, the converse will be the case. Recognizing the arrest of our knowledge at the point of the non-conversion of sugar into its subordinate elements, and finally into carbonic acid and water, (whether, with Barnard, we admit the conversion of the amyloid substance of the liver into sugar, or, with Pavy, believe that sugar is not converted into amaloid substance during life, and is subject to no transformation,) the aim must be both to lessen the source of the sugar and to promote the process of assimilation. *For the former we lessen the ingestion of sugar and starch—the chief sources of the sugar; and for the latter, increase the ingestion of nitrogen—the chief excitor, in the absence of exertion, of vital action.*

Hence the supply of the almost purely starchy foods, as arrowroot, tapioca, and sago, and the starchy foods poor in nitrogen, as rice and potatoes, should be entirely cut off, whilst the more highly nitrogenized vegetable foods, as wheaten flour, oatmeal, barleymeal, or ryemeal, must be used only within the narrowest limits. The highest nitrogenous foods of this class, as peas, beans, and lentils, if starchy food be given at all, should be eaten alone, or eaten with sharps—the inner husk of wheat. Beans in the whole scales should not be given, since only the inner part is digested, whilst the outer and by far the heavier portion passes off by the bowel; and hence bean, unless it could be taken in great quantities, can afford only an inconsiderable amount of nutriment.

Separated sugars have been given purposely as an article of food in diabetes; and why should they not be given? If the quantity of sugar excreted be increased only by the amount ingested, the chief effect has been the waste of so much food. The treatment is so far rather useless than injurious; but whether the mere absorption of so much useless matter, followed by its rapid excretion from the blood, interferes with other vital processes, and thereby does harm, is not known. If sugar act as a diuretic, its presence must be injurious; but if not, the evil which it does may be fully counteracted by the good effect of other components of the food with which it may be associated, as in the use of milk. *But sugar cannot be regarded as food when it is given to a diabetic patient*, and since it does exist in the urine, it is better that it should be produced from starch than given ready formed, since the transformation of the latter produces animal heat, whilst the mere absorption of sugar into the blood does hot increase heat. Well-fed flesh should be allowed, since the juices are rich in nitrogen, and the whole lean substance has much nitrogen in proportion to the carbon. In like manner, the preparation of gluten, albumen, aud gelatin, although containing about 40 per cent. of carbon, are the richest foods in the proportion of nitrogen to carbon. Skimmed milk, and butter-milk, and then new milk, rank next in this relation, and at the same time supply fluid.

*But with every attempt at limitation in the quantity of starch and sugar, a quantity of carbon and hydrogen must be allowed at least equal to that used in health*, or wasting will occur both from defect of ingesta and excess of egesta; and as I know, conditions allied to scurvy, and even death itself, may result, and have resulted, from this so-called remediable starvation. In a state of quietude in health not less than 9 oz. of carbon, and with ordinary exercise not less than 10 oz., must be supplied daily. These quantities, apart from the hydrogen, may be found in from 31 oz. to 35 oz. of bread, in about 13½ oz. to 15 oz. of fat, and in about 8 to 9 pints of new milk, and 9 to 10 pints of skimmed milk or butter-milk. It is very difficult to assign the equivalent in meat, since it depends upon the amount of fat contained in its juices, or laid up separately with the flesh, but ordinarily from 26 oz. to 29 oz. of meat would be required to furnish that amount of carbon. Hence it is easy to supply enough carbon for the production of animal heat from either separated or combined fats; and since, in the absence of starch, this is the chief source of the carbon which is converted into carbonic acid, we must trust to fat and oils as to a sheet anchor. The nitrogen to be supplied in diabetes, even when improvement has taken place, must not be less than 300 grains daily, and the quantity may be increased indefinitely, subject only to the limitation in the carbon which is associated in foods with it.

3. *Urea and salts.*—The quantity of these substances emitted must not be greater than the equivalent supply in the food, since otherwise they must be more abundantly derived from the tissues. Abundant nitrogen in food and a large emission of urea are proper. When, in the last stages of the disease, the digestion is very much weakened, a large part of the nitrogenous food will pass off by the bowel, and will not be converted into urea; and hence, with abundant food, the urea will be less than the equivalent nitrogen in the food.

4. *Animal heat.*—The production of animal heat is greatly lessened in diabetes, since so large a part of the healthy chemico-vitral transformations of food does not occur; and in order to maintain a due amount, it is essential that fat and other animal food capable of final transformation into carbonic acid should be continually given. The condition of the skin aids in lessening this evil, since diminution in the vaporization saves heat, and heat is chiefly lost by the less costly method of radiation. When sufficient woollen clothing is worn, and heat is supplied directly by hot food, the evil is proportionally lessened.

It may not appear philosophical to try to induce the conversion of sugar by secondary agencies, or the elimination of fluid by other organs than the kidneys, and by way of derivation; yet all the usual efforts to maintain health tend to that end: as, for example, exertion in the open air, which, through increase of the respiration, will increase the elimination of the final products of the conversion of sugar—carbonic acid and water, and by the increase of capillary action will tend to increase perspiration. Hence, exertion and free exposure to the air in the early periods of the disease are very important. The use of tea is also very beneficial, since it tends to increase both the respiratory and the cutaneous actions.

Thus, a summary of the proper diet in diabetes is as follows:

1. *Fluids.*—To be limited by degrees daily until they shall not exceed five pounds and a half in both fluid and solid food. Of this quantity two to three pints should consist of new or skimmed milk, and one pint, or less, of tea. In the cold season and at night they should always be given when hot. Of all alcohols brandy is the best, and may be given with water only, or added to milk, or beat up with egg and milk, and given several times daily. No fluid should be given in greater quantity than half a pint at a time, and when milk is reduced in volume by cooking, the daily quantity of fluid must be made up by an additional supply of the same or other fluid.

2. *Solids.*—Dr. Prout's combination of eggs and milk (with sharps substituted for bran) is excellent. Four ounces of sharps and 4 oz. of peas, beans, or lentils may be made into bread or pudding, with milk, or into omelettes with eggs and herbs.—Eggs and gelatin may be given when starchy food cannot be altogether intermitted. Eggs, gelatin, cheese, gluten bread, meat, fat, and oils may be given as largely as they can be digested. The free use of solid oil should be urged, whether in the cooking of fish or flesh, or in the use of water-cress as a solid, or drunk alone, so that several ounces may, if possible, be consumed daily; but as there are in all persons preferences and dislikes in reference to particular fats, that kind—whether butter, suet, oil, or fat of meat—should be allowed which is the most agreeable. Four oz. of sharps, 3 oz. of wheaten flour, 5 oz. of peas, 1 lb. of meat, 2 oz. of cheese, 2 pints of milk, and 3 eggs, will afford more than about 13 oz. of carbon and 1 oz. of nitrogen daily.—*London Lancet.*

